# Development of 3D Printable Silver Carp (*Hypophthalmichthys molitrix*) Surimi Gel with Dynamic High-Pressure Microfluidization-Modified Pea Protein Isolate and Microcrystalline Cellulose

**DOI:** 10.3390/foods13233966

**Published:** 2024-12-09

**Authors:** Xiaodan Liu, Qianyu Le, Yi Shi, Ying Yu, Jihao Zeng, Huiyun Chen, Jinhong Wu

**Affiliations:** 1 School of Food and Tourism, Shanghai Urban Construction Vocational College, Shanghai 201415, China; liuxiaodan@succ.edu.cn; 2 Department of Food Science and Engineering, School of Agriculture and Biology, Shanghai Jiao Tong University, No. 800 DongChuan Road, Minhang District, Shanghai 200240, China; qianyu.le@outlook.com (Q.L.);; 3 Institute of Agricultural Product Processing Research, Ningbo Academy of Agricultural Science, No. 19 Dehou Street, Yinzhou District, Ningbo 315040, China

**Keywords:** silver carp surimi, dynamic high-pressure microfluidization technique, pea protein isolate, microcrystalline cellulose, 3D printing

## Abstract

Sliver carp is a nutritious and abundant species in China, but its low market value stems from its thin meat, small bones and strong odor. Processing it into surimi enhances its economic value, though surimi typically has low gel strength and is prone to deterioration. Recently, three-dimensional (3D) printing has gained attention as an innovative additive manufacturing technique for personalization and process simplification requiring high-performance materials. This study intended to develop an optimized surimi formula for 3D printing with dynamic high-pressure microfluidization (DHPM)-modified pea protein isolate (PPI) and microcrystalline cellulose (MCC). Firstly, the effect of DHPM on PPI properties was evaluated, followed by the optimization of the surimi gel formula (72.093% water content, 3.203% PPI, 1.728% MCC, 1% salt, 1% collagen peptide and 20.976% sliver carp paste) and 3D printing parameters (2000 mm/min at 25 °C with a 1.5 mm nozzle). Rheological comparisons between the optimized surimi, surimi with commercial antifreeze and surimi with only PPI or MCC indicated that the optimized formulation exhibited clearer 3D printing outlines and reduced stickiness due to a higher recovery and lower loss modulus. These results demonstrated that DHPM-treated PPI and MCC enhanced the 3D printability of silver carp surimi gel, providing a new idea for a surimi product and supporting its potential applications in food 3D printing.

## 1. Introduction

China is one of the megadiverse freshwater regions around the world, with freshwater fish production contributing over 50% of the global total [1]. Sliver carp (*Hypophthalmichthys molitrix*) is a highly nutritious and abundant species in China. Currently, China’s freshwater fish market primarily focuses on live sales. Due to the thin meat, small bones and strong odors, silver carp holds a rather low market value. Thus, processing freshwater fish, such as silver carp, into surimi is a crucial method for increasing their economic value [2]. Surimi is a myofibrillar protein concentrate made from fish meat through processes including rinsing, chopping and crushing [3,4]. Surimi products offer benefits such as being low-calorie, low-fat, low-cholesterol and high-protein [5]. However, several issues limit their development. Freshwater fish surimi has low gel strength and is prone to gel deterioration. During freezing, proteins easily undergo denaturation, reducing viscoelasticity and the taste quality of surimi gel. Besides, freezing–thawing cycles lower the protein digestibility of surimi products [6]. Fish proteins can be classified into three types: myofibrillar (salt-soluble), sarcoplasmic (water-soluble) and matrix proteins (insoluble) [7]. Myofibrillar proteins (MPs) make up 65 to 80% of the total protein content and form elastic gels upon heating; therefore, elasticity is generally regarded as an indicator to evaluate the quality of surimi products [7]. Various additives, including non-meat proteins, inorganic salts, starch, polyphenols and hydrocolloids, are added to improve surimi quality and reduce the production costs.

Pea protein isolate (PPI) is a high-quality plant protein rich in essential and non-essential amino acids, low in fat and containing bioactive compounds [8]. Due to its nutritional value, low allergenicity and affordability, PPI is a promising non-meat protein additive [9,10]. Its incorporation improves the quality and health benefits of surimi gel [10]. Additionally, Sun et al. [11] illustrated that PPI could influence the rheological properties of surimi by altering protein–protein interactions. Besides, Moreno et al. [12] demonstrated that adding 1.41% PPI significantly increased viscoelasticity and preserved gel strength comparable to fresh surimi after one year of frozen storage. Microcrystalline cellulose (MCC) is a linear polysaccharide commonly used as a fat substitute, emulsifier, stabilizer and carrier [13,14]. Zhang et al. [15] indicated that additional cellulose inhibited water loss from low-salt surimi gels, and therefore enhanced surimi quality. Moreover, Mi et al. [16] stated that protein conformation was changed and a denser surimi gel network was formed by adding modified MCC. Therefore, PPI and MCC were chosen as additives to improve the quality of surimi gel in this study.

Three-dimensional (3D) printing, an innovative additive manufacturing technique, has gained widespread attention in the food industry due to its potential for personalized customization, a variety of edible materials and simplified processes [17,18,19]. For instance, Mirazimi et al. [20] developed a 3D printable puree with 0.2% agar for the elderly with dysphagia. Recently, Lu et al. [21] found that high internal phase emulsions, as filler, improved the 3D printability of surimi gels. Moreover, Zhu et al. [22] demonstrated that surface crosslinking using dry-spraying transglutaminase enhanced the 3D printability of surimi, establishing its potential as a transitional food for dysphagic individuals. 3D printing requires high-performance materials with specific attributes, such as extrudability, rheology and post-processing stability [18,23,24]. The behavior of food constituents during the printing process is critical for achieving high-quality structures. However, challenges persist in printing precise 3D shapes with food materials, which is a rather high requirement for 3D printable materials. Ideally, 3D printing materials should possess suitable flow properties for extrusion and stability after printing [25]. Surimi, a protein paste made of proteins, is a promising candidate for 3D printing, as modulating its composition, including proteins, carbohydrates, water content and fats, could significantly affect its properties and printability [26,27]. During 3D printing, the rheological properties of materials are crucial for extrusion, layer binding and support of deposited layers. However, the low solubility of PPI limits its emulsifying capacity, emulsion stability, foaming capacity, foaming stability and water/oil retention. Various studies have been conducted to enhance these characteristics. Dynamic high-pressure microfluidization (DHPM) is a versatile emerging technology that induces changes in protein structure depending on the nature of the molecules and processing parameters [28]. DHPM may improve or impair techno-functional properties of protein and its ability to bind other molecules, suggesting its potential to offer benefits for 3D printing materials, including minimal nutrient loss, absence of exogenous chemicals and reduced processing time [29,30]. Hu et al. [31] reported that microfluidization altered the conformational secondary structure of PPI, improving solubility and dispersibility. Similarly, Djemaoune et al. [32] found that microfluidized pea albumin aggregates exhibited enhanced foaming and emulsifying properties.

Despite extensive studies on 3D printed products, few reports address the formula of 3D-printable silver carp surimi gel. This study intended to develop an optimized surimi formula for 3D printing with modified PPI and MCC. Therefore, the effect of DHPM on the properties of PPI was firstly investigated. Then, the optimal surimi formula and 3D printing parameters were investigated to understand the correlation between the rheological properties of surimi gel and 3D printing behavior, guiding the further applications of 3D printed surimi gel.

## 2. Materials and Methods

### 2.1. Materials

Frozen sliver carp (*Hypophthalmichthys molitrix*) surimi was prepared at the Food Processing Laboratory, Shanghai Jiao Tong University. PPI and MCC were obtained from Shanghai Yuanye Biological Technology Co., Ltd. (Shanghai, China). Collagen peptide was sourced from GELITA (Liaoyuan) Gelatine Co., Ltd. (Liaoyuan, China). All chemicals (chromatographic or analytical grade) were purchased from Sinopharm Chemical Reagent (Shanghai, China).

### 2.2. Effect of DHPM on the Properties of PPI

#### 2.2.1. DHPM Treatment

PPI was dispersed in deionized water at 10% (*w*/*v*) and subjected to pressures of 40, 80, 120, 160 and 200 MPa using a high-pressure microfluidizer homogenizer (Maikefu Co., Ltd., Shanghai, China) to assess pressure effects. Additionally, the dispersion underwent 1 to 5 passes at 160 MPa to evaluate time effects. All samples were subsequently freeze-dried after DHPM.

#### 2.2.2. Chemical Properties

##### PPI Solubility

Solubility was measured based on the method from Yin [33]. Briefly, 50 mg of PPI was dispersed in 50 mL deionized water and stirred for 1 h. The mixture was then centrifuged at 5795× *g* for 20 min, and the protein content in the supernatant was determined using the Bradford method.

##### Zeta Potential of PPI

A 2 g/L pea protein solution was ultrasonicated for 10 min to achieve homogenization, followed by measurement with a zeta potential meter.

#### 2.2.3. Physical Properties

##### Raman Spectrometer

The secondary structure of protein was analyzed using a confocal micro-Raman spectrometer (inVia Qontor, Renishaw, Gloucestershire, UK) with an excitation wavelength of 785 nm and a 60 s integration time. Spectra were collected in the 500 to 3000 cm^−1^ range, and structure quantification was performed by WiRE 5.3 software.

##### Differential Scanning Calorimetry (DSC)

The thermal properties of PPI were measured by DSC (DSCTA 2910, TA Instruments, Wilmington, DE, USA). Approximately 5 mg PPI was sealed in an aluminum pan and heated from 20 to 180 °C at 10 °C/min. The peak temperature (T_p_) and the enthalpy change of denaturation (Δ*H*) were calculated using TRIOS 5.1 software.

##### Emulsifying Activity Index (EAI) and Emulsion Stability Index (ESI)

The EAI and ESI were measured using a modified version of a previously published method [34]. Briefly, 15 mL of 0.2% (*w*/*v*) PPI dispersion and 5 mL soybean oil were homogenized at 20,000 rpm for 60 s using a T25 homogenizer (IKA, Staufen, Germany). 100 μL of emulsion was collected at 0 and 10 min. After dilution in 0.1% (*w*/*v*) SDS solution, absorbance was measured at 500 nm using a UV–Vis spectrophotometer (Thermo Scientific, Madison, WI, USA). The EAI and ESI were calculated as follows:

(1) $$\mathrm{EAI}\;\left(\frac{m^{2}}{g}\right)=\frac{2\times 2.303\times N\times A_{0}}{10000\times L\times \varphi \times C}$$

(2) $$\mathrm{ESI}\;\left(\min\right)=\frac{A_{0}\times 10}{A_{0}-A_{10}}$$

where N is the dilution factor; A_0_ and A_10_ are the absorbance of diluted emulsions at 0 and 10 min, respectively; L is the optical path (1 cm); φ is the oil fraction; and C is the protein concentration (g/mL).

##### Foaming Capacity (FC) and Foam Stability (FS)

FC and FS were measured using a previously published method with minor modifications [35]. Briefly, 15 mL of 0.2% (*w*/*v*) PPI dispersion was homogenized at 20,000 rpm for 60 s using a T25 homogenizer (IKA, Germany). FC and FS were calculated as follows:

(3) $$\mathrm{FC}\left(\%\right)=\frac{V_{0}-15}{15}\times 100\%$$

(4) $$\mathrm{FS}\left(\%\right)=\frac{V_{0}-15}{V_{30}-15}\times 100\%$$

where V_0_ and V_30_ are the volume of dispersion at 0 and 30 min, respectively.

##### Water-Holding Capacity (WHC) and Oil-Holding Capacity (OHC)

The WHC and OHC of PPI were determined using a modified method from Yang [36]. Briefly, PPI (100 mg) was mixed with deionized water (2 mL) or soy bean oil (2 mL) and stirred continuously. After 30 min, the suspension was centrifuged at 8963× *g* for 15 min, and the supernatant and sediment were weighed. WHC and OHC were calculated using the following formula:

(5) $$\mathrm{WHC}=\frac{m_{2}-m_{0}}{m_{1}-m_{0}}$$

(6) $$\mathrm{OHC}=\frac{m_{2}-m_{0}}{m_{1}-m_{0}}$$

where m_2_ is the mass of the tube and sediment; m_1_ is the mass of the tube and PPI; and m_0_ is the mass of tube.

### 2.3. Investigating the Effect of Modified PPI on Surimi Gel

#### 2.3.1. Preparation of Surimi Gel

Silver carp (*Hypophthalmichthys molitrix*) was rinsed with iced water at a ratio of 1:5 (*w*/*v*) and repeated 2 to 3 times. The rinsed fish mince was placed in a chopper and ground for 3 to 5 min. Then, a low-salt phosphate buffer (0.05 M NaCl, pH 7.5) equivalent to 5 times the weight of the rinsed fish mince (*w*/*v*) was added, followed by homogenization for 1 min. The mixture was then centrifuged at 14,938× *g* for 15 min at 4 °C, and the precipitate was collected. The collected precipitate was mixed with a high-salt phosphate buffer (containing 0.6 M NaCl, pH 7.5) equivalent to 5 times the weight of the precipitate (*w*/*v*) and homogenized for 1 min. After allowing the mixture to stand in a 4 °C refrigerator for 1 h, it was centrifuged again at 14,938× *g* for 15 min at 4 °C to obtain the supernatant. Cold deionized water equivalent to 10 times the weight of the supernatant (*w*/*v*) was added to precipitate the myofibrillar proteins. The mixture was centrifuged at 14,938× *g* for 15 min at 4 °C, and the myofibrillar protein precipitate was collected.

Modified PPI (2.0%, 4.0%, 6.0%, 8.0% and 10.0%) was added to the myofibrillar protein precipitate with 1.0% collagen peptide as the antifreeze. Surimi samples containing 8.0% commercial antifreeze and those without any cryoprotectant were used as controls. Each sample was supplemented with 2.5% salt. The surimi proteins were processed into gels using a two-stage gelation method (40 °C for 1 h followed by 90 °C for 20 min). After gelation, the samples were cooled in iced water and stored at 4 °C. Rheological and physical properties (water distribution, microstructure, gel strength and color evaluation) were measured within 24 h.

#### 2.3.2. Physical Properties

##### Water Distribution

Water distribution was assessed by a low-field magnetic resonance imaging analyzer (MesoMR23-060H-I, Shanghai Newmax Electronic Technology Co., Ltd., Shanghai, China), operating at a proton resonance frequency of 21 MHz, with pulse widths of 12 μs (90°) and 24 μs (180°), a sampling frequency of 100 kHz, analog gain of 20 dB, cumulative repetitions of 4, and employing a CPMG (Carr–Purcell–Meiboom–Gill) sequence measurement.

##### Microstructure Observation

All samples were freeze-dried, sectioned into 5 × 5 × 5 mm^3^ cubes and gold-coated for 45 s prior to observation, and subsequently imaged under a scanning electron microscope (5 kV) (Sirion 200, FEI, Oregon USA).

##### Gel Strength

A universal TA texture analyzer (Shanghai Teng Ba Instrument Technology Co., Ltd., Shanghai, China) with a P/5S cylindrical probe was employed to measure gel strength at a pre- and post-test speed of 1.5 mm/s and a test speed of 0.5 mm/s over a distance of 1.0 mm [37]. Gel strength (GS) was calculated based on the formula below:

(7) $$\mathrm{GS}\;\left(g\cdot \mathrm{mm}\right)=F_{b}\times D_{d}$$

where F_b_ is the breaking force (g) and D_d_ is the deformation distance (mm).

##### Color Evaluation

Gel color was measured using an XD-1055 color photometer (Shanghai Modern Environment Engineering Technique Co., Ltd., Shanghai, China). Lightness (L*), redness/greenness (a*) and yellowness/blueness (b*) were quantified, with whiteness (W*) calculated as follows:

(8) $$W^{*}=100-\sqrt[{2}]{{\left(100-L^{*}\right)}^{2}+{a^{*}}^{2}+{b^{*}}^{2}}$$

#### 2.3.3. Rheological Properties

Rheological properties were assessed using a DHR1 rheometer (TA Instruments Ltd., Crawley, West Sussex, UK). Surimi gel samples were placed at the center of the carrier table, with a 1 mm slit, and 3-methylsilicone oil was applied around the sample to prevent water evaporation. Parameters were set as follows: temperature range 25 to 90 °C, heating rate 3 °C/min, strain 1% and angular frequency 10 rad/s [38]. Rheological properties of samples were quantified by the energy storage modulus (G′), loss modulus (G″) and loss coefficient (tanδ=G″/G′).

### 2.4. Response Surface Methodology (RSM) for Optimal Surimi Formula

The preliminary experiments identified water content (X_1_), PPI concentration (X_2_) and MCC (X_3_) as key factors influencing the printability and texture of surimi gel for optimizing 3D formulations using RSM. A Box–Behnken experimental design was developed using “Design Expert” 13.0 software, with factor levels as described in Table 1. Indicators were selected to evaluate 3D printability and texture of samples, including printing precision, printing stability, cooking stability, textural properties (spininess and resilience) and whiteness (as determined by the aforementioned methods). The weight coefficients were determined using the analytic hierarchy process (AHP), a multi-criteria decision-making method that integrates qualitative and quantitative judgments [39].

An extrusion-based 3D printer (Foodini, Natural Machines, Barcelona, Spain) was used to evaluate the 3D printability of samples. A cuboid test model (30 mm × 20 mm × 10 mm) was conducted, using a 1.5 mm diameter circular nozzle to extrude the samples on a plate at 25 °C. The volumes of printed products were measured using a Vernier caliper.

(9) $$\mathrm{Printing}\;\mathrm{precision}=\frac{V_{\mathrm{pp}}}{V_{m}}\;$$

(10) $$\mathrm{Printing}\;\mathrm{stability}=\frac{V_{\mathrm{pp}1}}{V_{m}}$$

(11) $$\mathrm{Cooking}\;\mathrm{stability}=\frac{V_{\mathrm{ppc}}}{V_{m}}$$

where V_pp_ is the volume of the printed product; V_m_ is the volume of the model; V_pp1_ is the volume of the printed product after 1 h; and V_ppc_ is the volume of the printed product cooked 10 min.

#### Texture Profile Analysis (TPA)

Texture properties were measured using a universal TA texture analyzer (Shanghai Teng Ba Instrument Technology Co., Ltd., Shanghai, China) with a P/36R cylindrical probe. The TPA testing protocol utilized a single mode with a pre- and post-test speed of 2.0 mm/s and test speed of 1.0 mm/s, with a trigger force of 5.0 g.

### 2.5. Processing Properties of Surimi Protein–PPI Composite Gel

#### 2.5.1. Formulations of Surimi Protein–PPI Composite Gel

Five formulas were designed to investigate the processing properties of surimi gels based on the optimized formulation from RSM results, as shown in Table 2.

#### 2.5.2. Rheological Properties of Surimi Protein–PPI Composite Gel

Rheological properties were assessed using a DHR1 rheometer (TA Instruments Ltd., Elstree, UK) with a 40 mm diameter plate geometry and a 1 mm gap, following a modified method based on Liu [40]. Shear-thinning properties were measured with a shear rate of 0.01 to 1000 s^−1^ and at 25, 30, 35 and 40 °C. Shear recovery properties were assessed at 25, 30, 35 and 40 °C. Samples were subjected to a low shear rate of 1 s^−1^ for 180 s, followed by a high shear rate of 100 s^−1^ for 120 s and then returning to 1 s^−1^ for 180 s. Recovery was quantified as the ratio of the viscosity (*η*) during the first 30 s of the third stage to the average *η* of the first stage. Self-supporting capacity was assessed at 25 °C, with a strain of 0.1% (in the linear viscoelastic region) and an angular frequency of 1 to 100 rad/s.

### 2.6. Optimized 3D Print Parameters for Surimi Protein–PPI Composite Gel

The print speed was set to 2500 mm/min at 25 °C, and samples were produced with different nozzle diameters (4.0, 1.5 and 0.8 mm) to assess their impact on print quality. Printing was conducted at a speed of 2500 mm/min with a nozzle diameter of 1.5 mm at various temperatures (25, 30, 35 and 40 °C) to assess temperature effects. Printing speeds were set as 1500, 2000, 2500, 3000 and 3500 mm/min (with a 1.5 mm nozzle and at 25 °C) to assess the effect of printing speed.

### 2.7. Statistical Analysis

All samples were measured at least in triplicate, and data are presented as mean values ± standard deviation (SD). Statistical significance was assessed by IBM SPSS 19 (*p* < 0.05).

## 3. Results and Discussion

### 3.1. Effect of DHPM on the Functionalities of PPI

**Figure 1:**
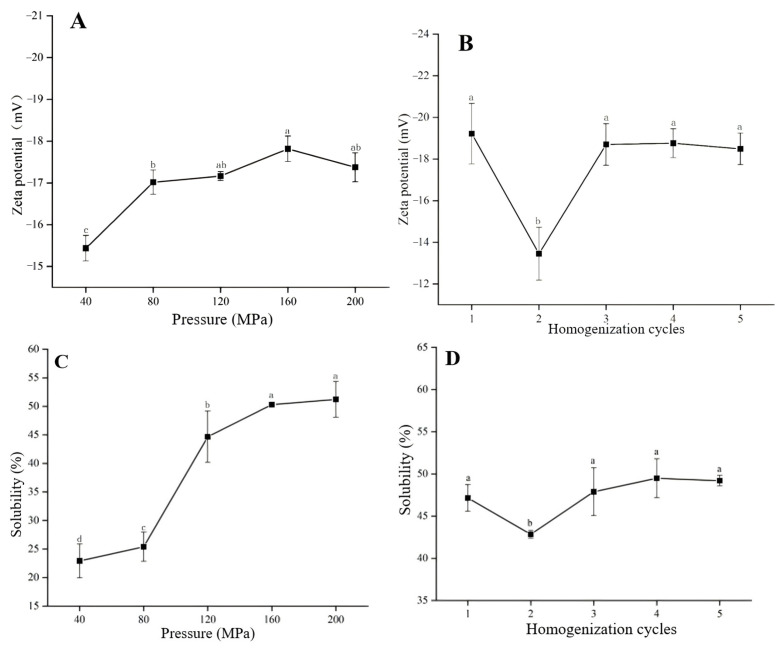
The effect of the DHPM parameter on the zeta potential and solubility of PPI: zeta potential was plotted on applied pressure (**A**) and on homogenization passes (**B**); solubility was plotted on applied pressure (**C**) and on homogenization passes (**D**); data with an identical letter indicate no significant difference between them (*p* > 0.05).

**Figure 2:**
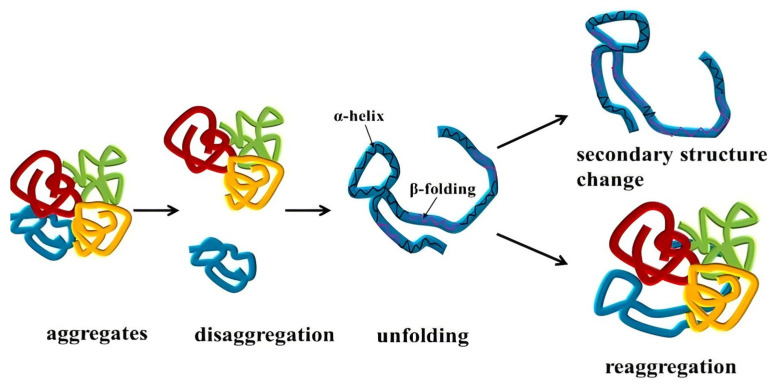
A possible schematic illustration of the structure change of protein during DHPM treatment.

Zeta potential and solubility of PPI exhibited a similar trend with increasing homogenization pressure, as shown in Figure 1, with the highest zeta potential and solubility observed at approximately 160 MPa. Zeta potential reflects the surface charge of suspended particles and, therefore, is a crucial indicator of colloidal dispersion stability [41]. The increased zeta potential suggested the enhanced stretching of the protein structure after DHPM treatment, exposing more negative charged amino acids [42]. However, with excessive pressure, zeta potential decreased, possibly due to protein over-stretching, causing PPI reaggregation via hydrophobic interactions, forming insoluble aggregates and re-embedding previously exposed amino acids [43]. Besides, DHPM treatment significantly improved PPI solubility by increasing the surface negative charge, with enhanced electrostatic repulsion between particles [41]. Solubility could serve as a reliable index for assessing protein functionality, reflecting protein denaturation and aggregation [44]. Figure 1 also illustrates the effect of homogenization cycles on zeta potential and solubility. After two homogenization cycles, both properties reached their lowest values, due to PPI aggregation and the embedding of amino acids on the protein surface. With more homogenization cycles, both properties returned to levels comparable to those observed after one cycle, due to the redispersion of protein aggregations caused by mechanical force induced by DHPM. With excessive DHPM pressure and cycles, both zeta potential and solubility decreased, possibly due to the reaggregation of PPI, as shown in Figure 2. The combination of hydrophobic interactions, hydrogen bonds, sulfhydryl/disulfide interchange reaction and electrostatic interactions contributed to the disaggregation and reaggregation of PPI [45]. Therefore, the optimized DHPM treatment conditions for PPI should be four homogenization cycles at 160 MPa.

Additionally, the functional, structural and thermal properties of PPI before and after DHPM treatment (four homogenization cycles at 160 MPa) were compared (Table 3), indicating that DHPM significantly improved PPI solubility and enhanced other characteristics, supporting its potential application in the food industry. The adsorption capacity of proteins at the oil–water interface was evaluated by the EAI and ESI. The interplay between hydrophilic and hydrophobic groups is crucial for the functional properties of proteins in emulsions and foams [46,47]. The optimized DHPM treatment increased all these values, possibly due to protein subunit unfolding [48], leading to greater exposure of hydrophilic and hydrophobic groups. Research indicated that appropriate high-pressure treatment enhanced the EAI and ESI of red kidney beans, while excessive pressure reduced these properties [33]. Li-Chan et al. [48] stated that solubility is the primary factor influencing the EAI of low-solubility proteins. The EAI and ESI initially increased and then decreased with increasing pressure, suggesting that moderate DHPM treatment improved PPI characteristics [33]. Moreover, Wang et al. [49] illustrated that the emulsion stability of DHPM-treated soy protein isolate increased significantly, due to the enhanced interface protein content, viscosity and viscoelasticity. As for WHC and OHC, not only protein conformation but also hydrophilic and hydrophobic balance could affect these values [46]. After DHPM treatment, WHC of PPI decreased, while OHC increased. The increased OHC was likely due to the unfolding of a protein unit, leading to the exposure of more hydrophobic groups [46,47]. Before DHPM, WHC was higher than OHC, possibly due to the higher hydrophilic amino acid (i.e., glutamic acid and aspartic acid) content of PPI [50]. This DHPM treatment might lead to crosslinking between protein molecules [51], resulting in the decreased WHC.

Raman spectroscopy and DSC data revealed the mechanism behind this enhancement. Raman spectroscopy technology has been widely applied to analyze the secondary structure of proteins [52]. The *α*-helix content increased, while the *β*-sheet, *β*-turn and random coil decreased, indicating significant conformational changes in PPI after DHPM treatment. This aligns with Chen’s findings [53], suggesting that DHMP treatment altered the secondary structure of proteins by promoting unfolding and aggregation. Moreover, Sun et al. [54] observed that the *α*-helix of zein decreased, while the random coil increased with the excessive DHMP pressure. As for thermal properties, DSC data confirmed that DHPM significantly increased both denaturation temperature (T_p_) and enthalpy (Δ*H*). The rise in T_p_ suggested structural alterations in PPI, while the higher Δ*H* indicated increased thermal stability due to the greater energy required for unfolding [55]. Therefore, moderate DHPM treatment significantly enhanced the functional properties of PPI by stabilizing its secondary structure.

### 3.2. Effect of Modified PPI on Surimi Gel

#### 3.2.1. Effect of Modified PPI on the Rheological Properties of Surimi Gel

**Figure 3:**
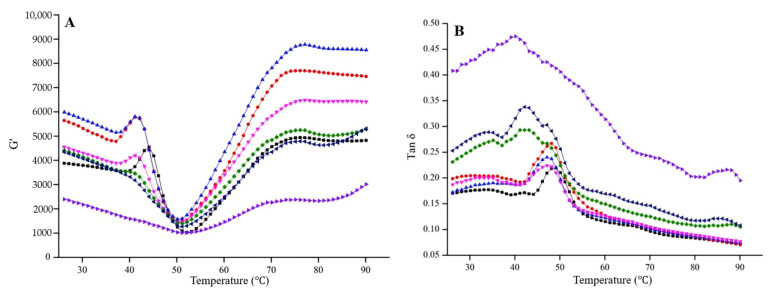
Effect of additional modified PPI on the G′ (**A**) and tan*δ* (**B**) of surimi gel; black line = gel with 8% commercial antifreeze (CA), red = surimi gel, light blue = gel with 2% PPI, pink = 4% PPI, green = 6% PPI, dark blue = 8% PPI and purple = 10% PPI.

The effect of additional modified PPI on the rheological properties of surimi gel is shown in Figure 3, where the energy storage modulus (G′) represents the elastic part of viscoelastic behavior (solid properties), the loss modulus (G″) describes the viscous part (liquid properties) and the loss coefficient (tan*δ*) quantifies the change in relative viscoelasticity of the gel during thermally induced gelation, with δ=0° for pure solids and 90° for pure liquids. Obviously, gel formed from 35 to 45 °C, leading to an increase in G′; while between 45 and 52 °C, gel weakening was observed, with a sharp decrease in G′, possibly due to (1) myosin degradation by endogenous protein hydrolases in fish muscle; (2) dissociation of actin–myosin and denaturation of myosin tails; and (3) hydrogen bond breakage from heating. Above 52 °C, the gel enhancement stage occurred, where G′ increased due to enhanced crosslinking between proteins and denaturation of myosin heavy chains and actin, resulting in thermally irreversible gels [56]. Generally, non-meat proteins serve as fillers, binders and bulking agents in meat products. Fillers are categorized as active or inactive: active fillers exhibit strong interactions with the substrate, leading to an increase in G′ with higher filler concentrations, while inactive fillers show minimal interaction, resulting in a decrease in G′ as filler quantity increases [57]. G′ decreased with increasing PPI addition due to the disruption caused by PPI to surimi protein interactions within the network, suggesting that PPI acted as an inactive filler in surimi gel. At lower concentrations, PPI provided structural support and, therefore, exhibited higher G′. However, at 10% PPI, competition for water between modified PPI and surimi protein hindered their interaction, resulting in decreased G′. Besides, during heating, G″ was consistently lower than G′ (tan*δ* < 1), indicating that the surimi gel was primarily elastic and exhibited good viscoelasticity. Increasing PPI content led to a gradual rise in G″, suggesting that the added protein reduced the elasticity of surimi gels, negatively affecting gelation. Similarly, Lee et al. [58] revealed that pea protein (≤ 60%) aggregates were dispersed within the continuous crosslinked surimi gel network, while above this concentration, the aggregates dominated, disrupting the network and forming coarse gels with larger pores. The viscoelasticity of surimi gels was comparable to that with commercial antifreeze (CA) only at 2% and 4% PPI addition.

#### 3.2.2. Effect of Modified PPI on the Water Distribution of Surimi Gel

T_2b_ and T_21_ denote tightly bound water in the sample, T_22_ indicates fixed water immobilized in the gel network and T_23_ refers to free water. As shown in Table 4, after adding 2% and 4% PPI, T_2b_ and T_21_ increased, while T_22_ decreased, indicating a shift of both bound and free water to fixed water. This suggests that PPI interfered with myofibrillar protein network formation, enhancing bound water mobility and absorbing free water, ultimately leading to increased water retention in coelacanth protein gel. As for higher PPI concentrations, T_2b_, T_21_, T_22_ and T_23_ decreased, approaching the values of surimi with 8% CA. This suggests that PPI might impair water mobility in surimi gel, possibly due to its competition for binding to water molecules.

#### 3.2.3. Effect of Modified PPI on Gel Strength and Microstructure of Surimi Gel

**Figure 4:**
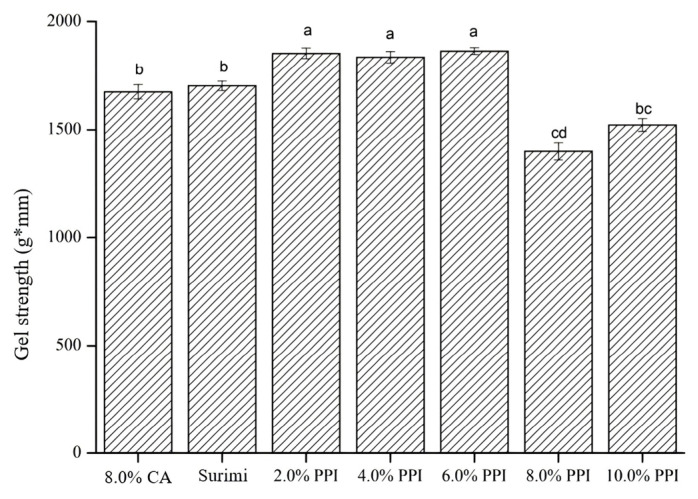
Effect of additional modified PPI on surimi gel strength; data with an identical letter indicate no significant difference between them (*p* > 0.05).

**Figure 5:**
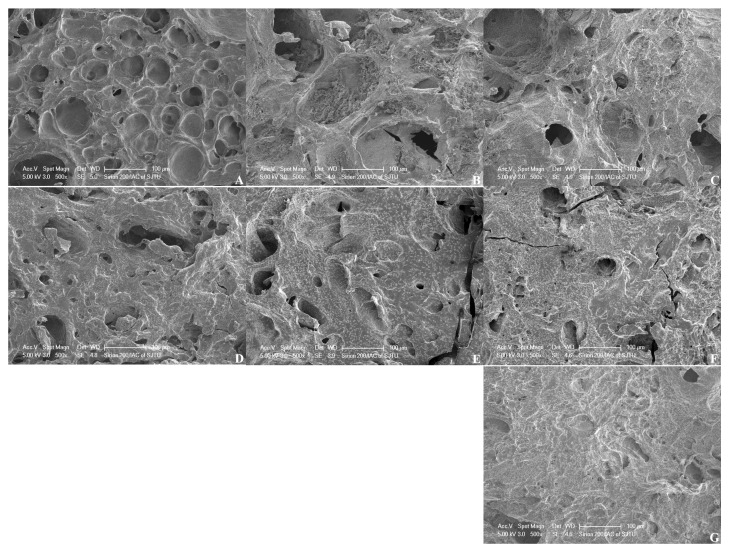
Effect of additional modified PPI on the microstructure of surimi gel: control (surimi gel) (**A**), surimi gel with 2% modified PPI (**B**), 4% PPI (**C**), 6% PPI (**D**), 8% PPI (**E**), 10% PPI (**F**) and with 8% commercial antifreeze (**G**).

As demonstrated in Figure 4, surimi gel strength was significantly improved after adding 2 to 6% PPI compared to the control and CA samples. Heat induces conformational changes in coeliac protein, facilitating the formation of a complex three-dimensional network through non-covalent crosslinking [59,60]. A more uniform and dense structure could enhance water retention and improve gel quality [61]. At lower concentrations, PPI was uniformly dispersed within the surimi gel network, acting as a filler, and thus enhancing gel strength. However, when PPI concentration exceeded 6%, gel strength decreased due to competition for water between PPI and surimi proteins, disrupting the surimi protein network formation [62]. Consistent with the microstructure observations as shown in Figure 5, surimi protein gels exhibited a robust gel network structure (Figure 5A); however, the addition of PPI progressively roughened the network, reducing void size and creating a more continuous and dense structure, indicating that PPI functioned merely as a filler instead of interacting with surimi protein. Besides, the overly dense structure might contribute to decreased elasticity and gel strength in surimi gels.

#### 3.2.4. Effect of Modified PPI on the Whiteness of Surimi Gel

As shown in Table 5, with the increasing PPI concentration, both whiteness and L* values decreased, while b* value increased. Park et al. [63] demonstrated that the color of surimi gel is influenced not only by surimi and exogenous additives but also by moisture content, which could enhance brightness and whiteness while reducing yellowness. Thus, the observed decrease in whiteness and brightness, along with the increase in yellowness, might result from the competition between PPI and surimi protein for moisture, leading to reduced surface moisture in the gel. Additionally, the inherent yellowish tint of PPI might also contribute to the increase in yellowness.

### 3.3. Optimized Surimi Formula for 3D Print

To optimize the formula of surimi protein–PPI composite gel, weights were assigned to each indicator based on their relative importance, with the consistency ratio (CR) = 0.061 (< 0.1), indicating that the judgment matrix for weight assignment was reasonable. The total score, calculated using the following formula according to Appendix A, Table A1, was utilized for further evaluation in RSM:

$$\begin{array}{cc} \mathrm{Total}\;\mathrm{score}= & 0.332\times \mathrm{printing}\;\mathrm{precision}+0.17068\times \mathrm{printing}\;\mathrm{stability}\\ & \;+0.21427\times \mathrm{cooking}\;\mathrm{stability}+0.10457\times \mathrm{gel}\;\mathrm{strength}\\ & \;+0.07859\times \mathrm{springiness}+0.07859\times \mathrm{resilience}\\ & \;+0.03129\times \mathrm{whiteness} \end{array}$$

RSM experiments and results are demonstrated in Appendix A, Table A2. The relationship between moisture content (X_1_), modified PPI concentration (X_2_) and MCC concentration (X_3_) on the total score response, as determined through regression analysis, could be expressed by the following function (the statistical model was reasonable and reliable according to the analysis of variance in Appendix A, Table A3):

$$\begin{array}{cc} \mathrm{Total}\;\mathrm{score}\;\left(Y_{1}\right) & =299.38-50.40X_{1}+3.57X_{2}-3.03X_{3}-0.0781X_{1}X_{2}\\ & \;+4.50X_{1}X_{3}-27.52X_{2}X_{3}-57.44X_{1}^{2}-29.48X_{2}^{2}-39.96X_{3}^{2} \end{array}$$

**Figure 6:**
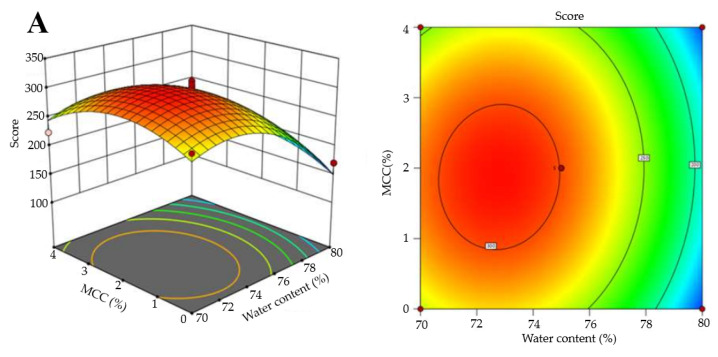

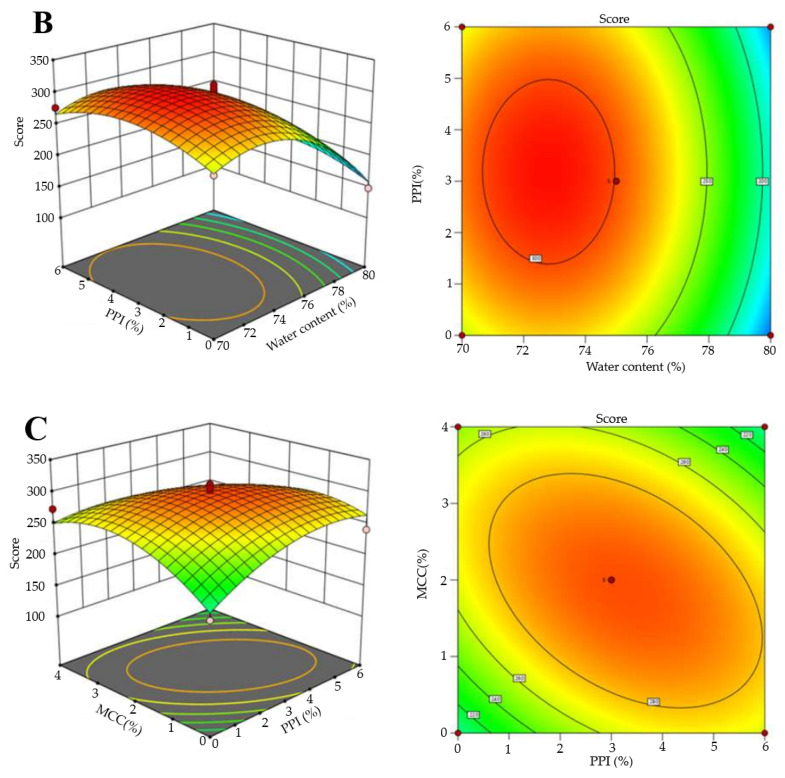
Three-dimensional figures from RSM based on weight percentages: the interactive effects of water content, PPI and MCC concentration on the properties of surimi gels. (**A**): Interactive effects of water content and MCC on gel properties; (**B**): Interactive effects of water content and PPI on gel properties and (**C**): Interactive effects of PPI and MCC on gel properties.

As for RSM, interaction between factors could be indicated by the contour line shape: circular for non-significant interactions and elliptical for significant interactions. Figure 6 demonstrates the response results under various factor combinations. The interaction between water content and PPI or MCC exhibited a nearly circular contour, suggesting there was no significant interaction. In contrast, the elliptical contour lines for the interaction between PPI and MCC indicated their interactions. Moreover, at a constant concentration of PPI or MCC, the total score increased with decreasing water content, due to the competition for water between PPI or MCC and surimi protein. At constant PPI and water content, the total score first increased, then decreased with increasing MCC. With constant MCC and water content, the total score exhibited the same result as PPI increased. Additional PPI and MCC interacted and acted as fillers in surimi gel, improving gel quality, while excessive amounts led to competition for water among PPI, MCC and surimi proteins.

The model predicted the optimal formula (total score = 310.87) as 72.093% water content, 3.203% PPI, 1.728% MCC, 1% salt, 1% collagen peptide and 20.976% sliver carp paste. Experimentally, the total score of this optimal formula was 320.5, suggesting the reliability of model.

### 3.4. Processing Properties of Optimized Surimi Protein–PPI Composite Gel

Materials experience shear stress changes during extrusion from the nozzle to the print platform, making rapid recovery from high shear critical for the structural stability of 3D printed products [64]. An optimal 3D printable material should exhibit both shear thinning and fast recovery. Shear thinning enables smooth extrusion, while fast recovery allows the material to quickly regain viscosity and mechanical properties, ensuring structural stability [65,66].

#### 3.4.1. Rheological Properties of Optimized Surimi–PPI Composite Gel

##### Shear-Thinning Behavior

**Figure 7:**
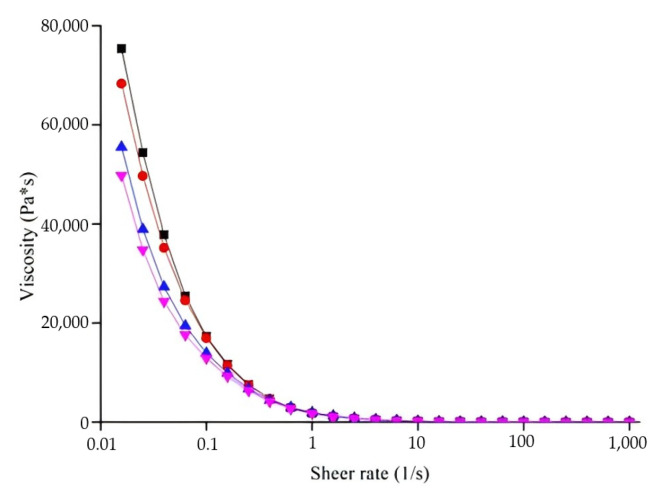
Shear-thinning behavior of surimi gels: viscosity plotted on the shear rate at 25 °C, with different colors representing different gel compositions (black = optimized gel formula; red = the control; blue = gel with only MCC; pink = gel with only PPI).

The viscosities of samples decreased significantly with increasing shear rate at 25 °C, as illustrated in Figure 7, indicating pseudoplastic, shear-thinning behavior in all samples. This might be attributed to the deformation of surimi–PPI gel under shear (e.g., extension, dispersion), reducing flow resistance [67]. The pressure needed for continuous flow depends primarily on the viscosity and shear-thinning behavior of materials [68]. Viscosity (*η*) influences both the extrudability and shape stability of the sample. Lower viscosities ease extrusion, while higher viscosities improve water retention, reducing slumping [69]. Therefore, as one of the important parameters, viscosity has to satisfy a certain value to extrude from the diameter of the nozzle and be stackable with deposited layers [70]. Interactions between surimi protein and PPI, protein–water binding and molecular diffusion could influence the viscosity of surimi–PPI gel. Such a shear-thinning property reduced material viscosity under shear force, aiding extrusion. Viscosity of gels containing only MCC or PPI was lower than the control, while the optimized formulation exhibited higher viscosity (Figure 7). This reduction in viscosity might result from the deformation of MPs, involving changes in orientation, stretching, and/or dispersion [70]. MPs are the major constituent of gel formation in surimi and play an important role in the processing characteristics of surimi products [71]. Proteins can interact in various ways: compatible, semi-compatible or incompatible [34]. In compatible systems, proteins form a dense network, while in incompatible systems, one protein may act as a filler in the network of other proteins. Sun et al. [11] reported that PPI disrupted MP interactions due to PPI or MCC incompatibility with MPs, reducing surimi viscosity. However, the simultaneous addition of PPI and MCC (the optimized formula) increased viscosity, possibly due to their combined effects with MPs, warranting further investigation.

##### Shear Recovery Behavior

**Figure 8:**
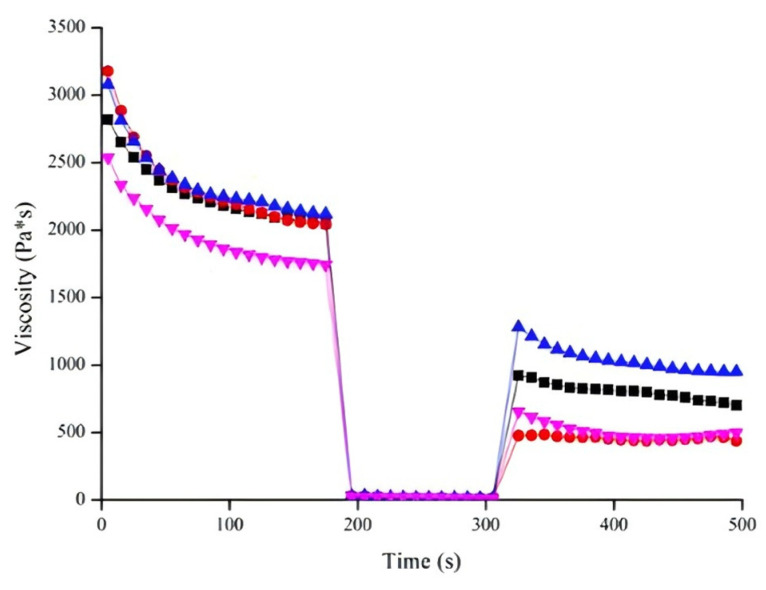
Shear recovery behavior of surimi gels: viscosity plotted on the shear time at 25 °C, with different colors representing different gel compositions (black = optimized gel formula; red = the control; blue = gel with only MCC; pink = gel with only PPI).

**Figure 9:**
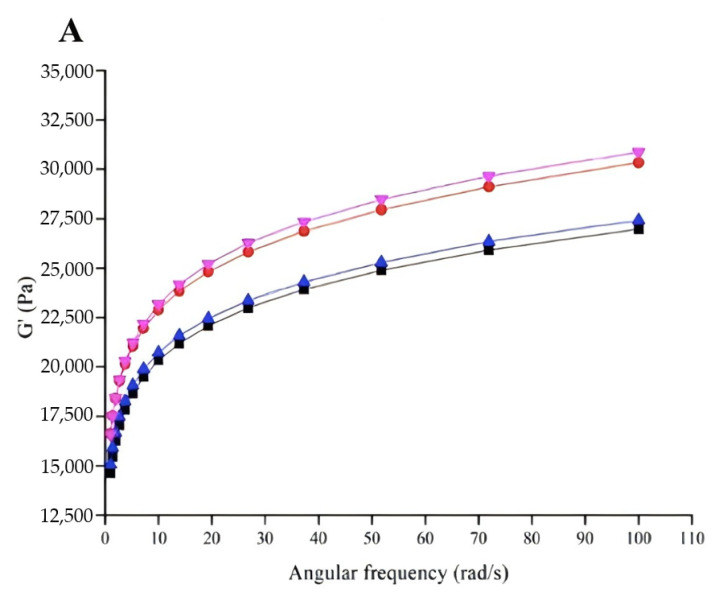

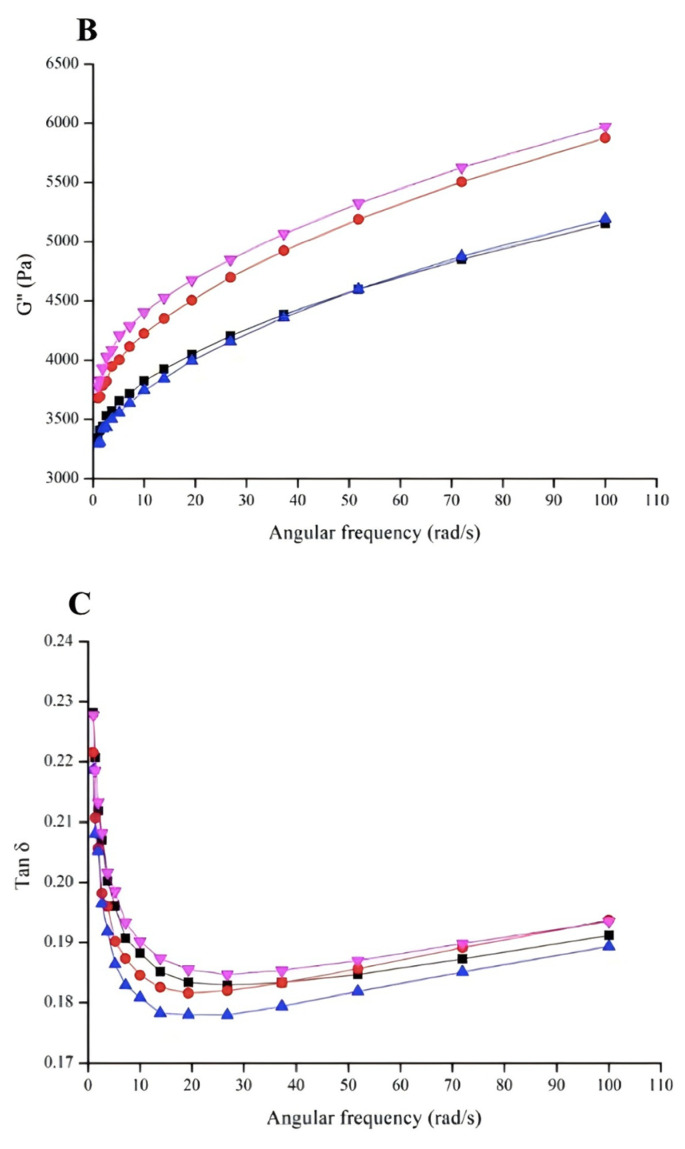
Dynamic rheological properties of surimi gels at 25 °C: rheological properties on angular frequency: (**A**) for the storage modulus (G′), (**B**) for the loss modulus (G″) and (**C**) for tan*δ*, with different colors representing different gel compositions (black = optimized gel formula; red = the control; blue = gel with only MCC; pink = gel with only PPI).

After extrusion of gel from the nozzle to the printing platform, shear force rapidly decreases. A fast, reversible viscosity response after high extrusion rates helps the material regain sufficient mechanical strength to support the next layer [65]. Therefore, shear recovery is crucial for the structural stability of 3D printed products [72]. Shear recoverability was assessed by the ratio of viscosity in the first 30 s of the third stage to the average viscosity of the first stage. Figure 8 demonstrates shear recovery behavior under alternating shear rates. Recovery percentages for the optimized formula, control, MCC-only gel and PPI-only gel were 39.16%, 20.47%, 50.51% and 30.74%, respectively, indicating that adding PPI or MCC enhanced the reversible structural behavior of surimi gel. Combined with the viscosity data, interaction between MPs and PPI might indicate that PPI primarily acted as a filler. This incompatibility likely reduced viscosity under shear, while additional PPI, acting as a filler, might accelerate the structural recovery of surimi gel. Moreover, as shown in Figure 9, both G′ and G″ decreased with the addition of PPI at a given oscillatory frequency, possibly due to PPI disrupting protein–protein interactions in MPs. Conversely, G′ and G″ increased with the addition of MCC, consistent with previous studies showing that MCC enhanced G′ and G″ in meat batters [73]. Thus, MCC could be used as a thickener to create a denser gel network.

##### Self-Supporting Capability

As shown in Figure 9, G′ values exceeded G″, indicating the self-supporting ability of surimi gel under external force, which might be helpful to maintain the structure after 3D printing [74], since 3D printing also requires material to be mechanically strong enough to maintain its structural stability [65]. This is primarily due to the entanglement of surimi protein molecules, forming a gel network. The simultaneous addition of PPI and MCC reduced the G′ and G″ of surimi gel by embedding as a filler in the surimi protein network, hindering crosslinking between surimi molecules, and thus decreasing gel viscoelasticity.

#### 3.4.2. Processing Characteristics

**Figure 10:**
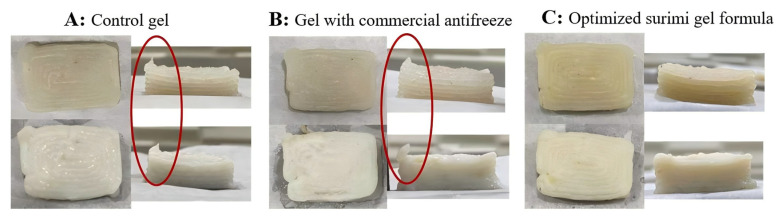
Representative images of 3D printed surimi gels: control group (**A**); surimi with 8% commercial antifreeze (**B**) and optimized surimi gel formula (**C**), with red circles indicating 3D printing defects.

All tested samples in this study could be regarded as pseudoplastic fluids with shear-thinning behavior, and therefore were deemed suitable for practical applications of extrusion-based 3D printing [65]. Table 6 illustrates the processing characteristics of surimi gels. The optimized formula exhibited slightly higher printing precision compared to the control, while printing stability, cooking stability and gel strength of the optimized formula were significantly improved, suggesting that PPI and MCC enhanced the stability and quality of surimi gel. These findings aligned with the RSM results. During 3D printing (Figure 10), the optimized formula performed clearer outlines than the control and was less prone to sticking, consistent with reduced G″ and improved recovery. This aligns with previous research, indicating that 3D printable surimi gels exhibit shear-thinning and solid-like behavior, while higher recovery and lower G″ contribute to better line clarity and reduced sticking [74].

### 3.5. Optimization of 3D Print Parameters

#### 3.5.1. Nozzle Diameters

**Figure 11:**
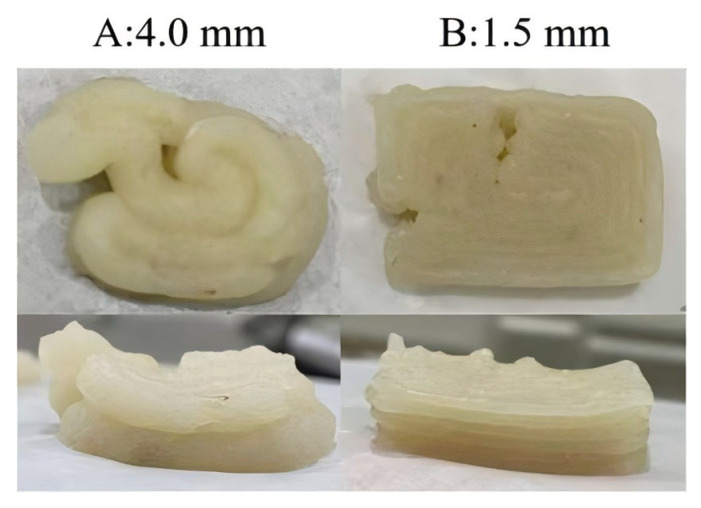
Representative pictures of 3D printed surimi gels with different nozzle diameters: 4.0 mm (**A**) and 1.5 mm (**B**).

As shown in Figure 11, a 4.0 mm nozzle produced thicker extrusion lines (Figure 11A), reducing accuracy and increasing roughness, while a 0.8 mm nozzle caused material breakage, leading to print failure. Nozzle diameter is crucial for print accuracy and product refinement. The smaller diameter enhances accuracy and product details; however, excessively small nozzles might cause material extrusion issues, increasing the risk of stripe breakage and printing failures [66]. To optimize printing accuracy and surface refinement, a 1.5 mm nozzle diameter was ideal, as it allowed smooth extrusion and clear outlines (Figure 11B). Similarly, Wang et al. [26] indicated that the 2.0 mm nozzle was optimal for 3D printing surimi products.

#### 3.5.2. 3D Printing Temperature

**Figure 12:**
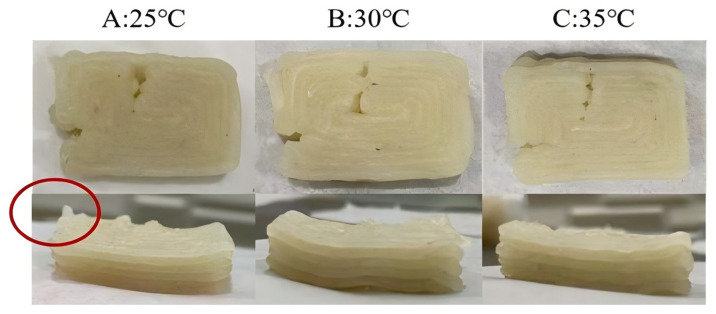
Representative pictures of 3D printed surimi gels at different temperatures: 25 °C (**A**), 30 °C (**B**) and 35 °C (**C**), with the red circle marking 3D printing defects.

As shown in Figure 12, 3D printing accuracy was significantly influenced by temperature, probably because of the effect of temperature on the viscosity of surimi gel [65]. At 40 °C, surimi gel exhibited a solid-like nature due to a marked viscosity reduction, rendering it unsuitable for extrusion and printing. As illustrated in Figure 12, between 25 and 35 °C, surimi gel was easily extruded, with viscosity decreasing as temperature increased, leading to reduced adhesion between printed lines and improved accuracy. However, at 30 to 35 °C, prolonged printing induced protein denaturation, gradually causing solidification and making it unsuitable for extended printing. Therefore, the optimal 3D printing temperature should be 25 °C despite slight printing defects.

#### 3.5.3. 3D Printing Speed

**Figure 13:**
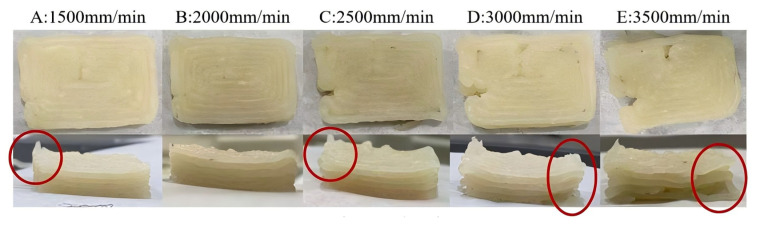
Representative pictures of 3D printed surimi gels using different speeds:1500 mm/min (**A**), 2000 mm/min (**B**), 2500 mm/min (**C**), 3000 mm/min (**D**) and 3500 mm/min (**E**), with red circles marking 3D printing defects.

One major bottleneck in the industrialization of 3D printing technology is its low efficiency, often addressed by increasing printing speed or nozzle diameter. However, a larger nozzle diameter compromises product detail [75,76]. Thus, printing speed is typically increased to enhance efficiency. As shown in Figure 13, speeds of 2500 to 3500 mm/min led to broken stripes, with higher speeds worsening the issue. The highest accuracy occurred at 1500 mm/min, but slow extrusion resulted in slight line adhesion due to the shear-thinning properties of surimi gels. At 2000 mm/min, accuracy was comparably high and the outlines were smooth with no noticeable adhesion, making it the optimal 3D printing speed for surimi gels.

### 3.6. Demonstration of Final Products

**Figure 14:**
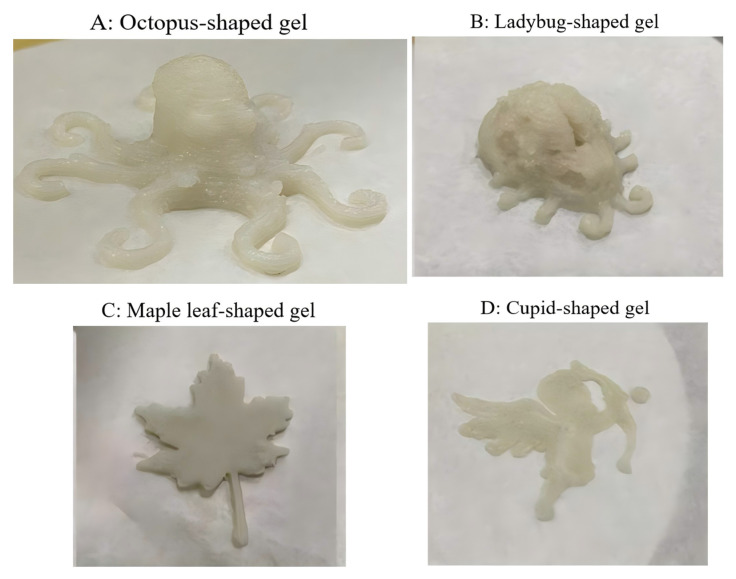
3D printed samples using the optimized surimi–PPI composite gel: octopus shaped (**A**), ladybug-shaped (**B**), maple leaf-shaped (**C**) and cupid-shaped (**D**).

Samples with different shapes were printed using the optimal 3D printing parameters (2000 mm/min at 25 °C with a 1.5 mm nozzle) and formula (surimi gel with 3.2% PPI and 1.7% MCC). As demonstrated in Figure 14, samples exhibited high resolution, well-defined 3D structure and a smooth surface, suggesting the potential of surimi–PPI composite gel in 3D printing for personalized customization.

## 4. Conclusions

This study first investigated the effect of DHPM on the properties of PPI, indicating that the optimum DHPM treatment parameters were four passes of homogenization at 160 MPa. This approach not only increased the solubility of PPI by increasing the surface negative charge, thus improving its characteristics, but also increased its regular structure to improve its overall stability. Moreover, the optimal surimi gel formula was developed via RSM as 72.093% water content, 3.203% PPI, 1.728% MCC, 1% salt, 1% collagen peptide and 20.976% sliver carp paste. This formula outperformed others regarding its printing precision, printing stability, cooking stability, springiness, resilience and whiteness. For 3D printing, materials with shear-thinning and solid-like behavior are ideal; thus, the rheological properties of surimi gels were evaluated, indicating that adding DHPM-treated PPI and MCC improved the 3D printability of surimi gel. The optimized surimi gel exhibited better 3D printed outline clarity and reduced sticking due to its higher recovery and lower G″. The addition of PPI and MCC significantly influenced the shear-thinning behavior, viscosity, recovery percentage and self-supporting ability of surimi gel due to their incompatible interactions with MPs. This incompatibility reduced viscosity, improving the extrusion process and minimizing sticking post-printing. PPI, as a filler, enhanced recovery percentage, leading to surimi gel with better printability. MCC compensated for the interference of PPI with MPs, promoting a denser gel network that improved self-supporting properties. Furthermore, optimal 3D printing parameters were investigated, indicating that the optimal 3D printing parameters were 2000 mm/min at 25 °C with a 1.5 mm nozzle. Overall, this study indicated that PPI and MCC were capable of improving the 3D printing behavior of silver carp surimi gel and successfully developed the optimal surimi formula with optimized 3D printing parameters, paving the way for broader applications of surimi gel in food 3D printing, including tailored foods for dysphagic patients, personalized nutrition and biomimetic aquatic products.

## Figures and Tables

**Table 1:** The Box–Behnken experimental design based on the weight percentage of water content, PPI and MCC concentrations.

Factor Levels Figure 1	Water Content X_1_ (%)	PPI X_2_ (%)	MCC X_3_ (%)
−1	70	0	0
0	75	3	2
1	80	6	4

**Table 2:** Designed formulation for comparing the processing properties of different surimi gels based on weight percentage.

Group Label	Additional Antifreeze	Water Content (%)	PPI (%)	MCC (%)	Salt (%)
Optimized	1% collagen peptide	72	3.2	1.7	1
Commercial	8% commercial antifreeze	72	0	0	1
Control	1% collagen peptide	72	0	0	1
Only PPI	1% collagen peptide	72	3.2	0	1
Only MCC	1% collagen peptide	72	0	1.7	1

**Table 3:** Comparison of PPI functional, structural and thermal properties before and after DHPM treatment (four homogenization cycles at 160 MPa).

PPI Properties	Before DHPM	After DHPM
Functional properties		
Solubility (%)	16.98 ± 0.34 ^b^	47.08 ± 5.56 ^a^
EAI (m^2^/g)	82.14 ± 0.81 ^b^	103.87 ± 1.47 ^a^
ESI (min)	10.06 ± 0.05 ^b^	13.28 ± 1.62 ^a^
FA (%)	47.78 ± 1.92 ^b^	88.33 ± 8.10 ^a^
FS (%)	27.94 ± 1.10 ^b^	83.82 ± 13.52 ^a^
WHC (g/g)	9.17 ± 0.70 ^a^	6.08 ± 1.65 ^b^
OHC (g/g)	4.62 ± 0.01 ^b^	5.83 ± 0.88 ^a^
Structural properties		
***α***-helix (%)	23.51 ± 5.92 ^b^	33.67 ± 1.87 ^a^
***β***-sheet (%)	43.75 ± 4.54 ^a^	35.97 ± 1.43 ^b^
***β***-turn (%)	20.16 ± 0.93 ^a^	18.57 ± 0.29 ^b^
random coil (%)	12.21 ± 0.36 ^a^	11.59 ± 0.11 ^b^
Thermal properties		
***T_p_*** (℃)	86.43 ± 1.17 ^b^	88.39 ± 0.29 ^a^
**Δ*H*** (J/g)	210.28 ± 2.67 ^b^	222.23 ± 0.82 ^a^

Data with identical letters indicate no significant difference between them (*p* > 0.05).

**Table 4:** Comparison of water distribution in different surimi gels.

Group Label	T_2b_ (ms)	T_21_ (ms)	T_22_ (ms)	T_23_ (ms)
8% commercial antifreeze	0.26 ± 0.06 ^c^	4.09 ± 0.93 ^a^	75.65 ± 0.00 ^a^	628.03 ± 118.25 ^ab^
Surimi	0.25 ± 0.05 ^c^	4.06 ± 0.08 ^ab^	72.34 ± 1.38 ^b^	631.17 ± 55.01 ^ab^
2% PPI	1.30 ± 0.21 ^a^	4.51 ± 0.11 ^a^	61.06 ± 5.58 ^c^	633.59 ± 102.81 ^a^
4% PPI	0.53 ± 0.04 ^b^	4.75 ± 0.00 ^a^	54.62 ± 0.00 ^b^	480.22 ± 46.29 ^c^
6% PPI	0.28 ± 0.04 ^c^	3.43 ± 0.00 ^a^	54.62 ± 0.00 ^cd^	580.85 ± 66.72 ^ab^
8% PPI	0.23 ± 0.28 ^c^	4.75 ± 0.00 ^a^	54.62 ± 0.00 ^cd^	493.49 ± 56.57 ^ab^
10% PPI	0.23 ± 0.28 ^c^	4.04 ± 0.00 ^ab^	50.52 ± 5.80 ^d^	327.46 ± 0.00 ^d^

Data with identical letters indicate no significant difference between them (*p* > 0.05).

**Table 5:** Effect of modified PPI on the color of surimi gel: L* represents the brightness of the sample (L* = 0 signifies maximum darkness); a positive a* value indicates red, and negative indicates green; positive b* values indicate yellow, and negative indicate blue.

Group Label	L*	a*	b*	Whiteness
8% commercial antifreeze	73.13 ± 0.60 ^c^	−0.40 ± 0.25 ^e^	3.89 ± 0.37 ^e^	72.84 ± 0.62 ^bc^
Surimi	76.30 ± 0.47 ^a^	0.80 ± 0.01 ^a^	5.86 ± 0.33 ^d^	75.59 ± 0.39 ^a^
2% PPI	74.67 ± 0.56 ^b^	0.77 ± 0.19 ^a^	6.29 ± 0.26 ^d^	73.88 ± 0.56 ^b^
4% PPI	73.30 ± 0.49 ^c^	0.48 ± 0.11 ^b^	6.99 ± 0.18 ^c^	72.39 ± 0.47 ^c^
6% PPI	72.94 ± 0.26 ^cd^	0.32 ± 0.17 ^bc^	7.45 ± 0.79 ^bc^	71.92 ± 0.44 ^c^
8% PPI	72.66 ± 0.90 ^cd^	0.18 ± 0.16 ^c^	7.79 ± 0.39 ^b^	71.57 ± 0.88 ^cd^
10% PPI	71.67 ± 2.04 ^d^	−0.12 ± 0.22 ^d^	8.65 ± 0.55 ^a^	70.83 ± 2.10 ^d^

Data with identical letters indicate no significant difference between them (*p* > 0.05).

**Table 6:** Processing characteristics (3D printing precision, stability, cooking stability, gel strength, springiness, resilience and whiteness) of surimi gels (the control, with commercial antifreeze, MMC, PPI and the optimized formula).

Group Label	Printing Precision (%)	Printing Stability (%)	Cooking Stability (%)	Gel Strength (g·mm)	Springiness	Resilience	Whiteness
Control	96.44 ± 1.78 ^b^	92.94 ± 1.84 ^c^	72.25 ± 0.01 ^b^	1052.95 ± 21.64 ^c^	0.87 ± 0.01 ^a^	0.20 ± 0.02 ^c^	71.33 ± 0.41 ^a^
Commercial Antifreeze	96.09 ± 2.28 ^ab^	94.42 ± 0.01 ^b^	71.33 ± 4.04 ^b^	1500.23 ± 8.48 ^b^	0.88 ± 0.03 ^a^	0.22 ± 0.01 ^b^	69.54 ± 0.48 ^b^
Optimized formula	98.85 ± 0.46 ^a^	96.00 ± 1.20 ^a^	88.21 ± 2.41 ^a^	2401.81 ± 16.37 ^a^	0.87 ± 0.04 ^a^	0.23 ± 0.01 ^a^	68.43 ± 1.38 ^b^

Data with identical letters indicate no significant difference between them (*p* > 0.05).

## Data Availability

The original contributions presented in this study are included in the article. Further inquiries can be directed to the corresponding author.

## Appendix A

The weight of each indicator was determined based on its relative importance using SPSS AU 24.0 software, where CR = 0.061, < 0.1, confirming the consistency of the evaluation judgment matrix. The total score was calculated according to the weight values in Table A1.

**Table A1:** Table of analytic hierarchy process (AHP) weight coefficients for RSM.

Indicator	Eigenvector	Weight	CI	RI	CR
Printing precision	2.254	32.200%	0.083	1.360	0.061
Printing stability	1.195	17.068%			
Cooking stability	1.500	21.427%			
Gel strength	0.732	10.457%			
Springiness	0.550	7.859%			
Resilience	0.550	7.859%			
Whiteness	0.219	3.129%			

The response surface experiment results were weighted and scored based on the obtained calculation formula as shown in Table A2.

**Table A2:** RSM experiment design and results.

Code	Printing Precision (%)	Printing Stability (%)	Cooking Stability (%)	Gel Strength (g·mm)	Springiness	Resilience	Whiteness	Total Score
1	96.05 ± 0.21	90.74 ± 0.06	87.64 ± 0.04	988.44 ± 32.13	0.83 ± 0.01	0.17 ± 0.01	68.11 ± 0.46	170.77
2	93.71 ± 0.11	87.18 ± 0.12	87.43 ± 0.10	1827.09 ± 24.32	0.92 ± 0.01	0.23 ± 0.01	67.24 ± 0.10	257.04
3	92.22 ± 0.10	86.31 ± 0.09	87.43 ± 0.11	2302.88 ± 12.42	0.81 ± 0.02	0.22 ± 0.01	68.92 ± 0.28	306.21
4	90.87 ± 0.34	83.18 ± 0.11	83.20 ± 0.05	2009.96 ± 14.23	0.86 ± 0.01	0.24 ± 0.01	67.81 ± 0.61	273.67
5	92.26 ± 0.31	90.74 ± 0.14	90.74 ± 0.21	1968.16 ± 21.23	0.81 ± 0.01	0.17 ± 0.00	69.77 ± 0.40	272.71
6	97.65 ± 0.41	88.45 ± 0.10	92.40 ± 0.13	1172.92 ± 11.03	0.89 ± 0.01	0.24 ± 0.01	68.93 ± 0.14	191.24
7	94.42 ± 0.11	76.12 ± 0.21	83.18 ± 0.17	1435.60 ± 10.38	0.89 ± 0.01	0.11 ± 0.01	69.20 ± 0.30	213.58
8	90.74 ± 0.12	83.57 ± 0.13	90.74 ± 0.20	1971.41 ± 14.94	0.90 ± 0.02	0.25 ± 0.01	68.52 ± 0.42	271.31
9	92.22 ± 0.08	80.54 ± 0.09	84.50 ± 0.05	993.80 ± 16.83	0.74 ± 0.01	0.10 ± 0.01	69.58 ± 0.31	167.71
10	97.65 ± 0.10	83.70 ± 0.10	91.50 ± 0.03	1660.69 ± 21.03	0.86 ± 0.01	0.22 ± 0.01	68.67 ± 0.50	241.23
11	96.92 ± 0.19	97.67 ± 0.17	90.74 ± 0.14	2247.79 ± 9.04	0.84 ± 0.01	0.12 ± 0.01	67.74 ± 0.43	304.57
12	96.62 ± 0.22	96.79 ± 0.21	95.14 ± 0.07	1473.06 ± 16.39	0.85 ± 0.01	0.16 ± 0.01	65.34 ± 0.31	224.18
13	96.07 ± 0.14	89.08 ± 0.14	92.74 ± 0.21	688.42 ± 5.03	0.93 ± 0.01	0.24 ± 0.01	68.94 ± 0.38	140.25
14	96.05 ± 0.21	90.55 ± 0.02	95.65 ± 0.18	2328.88 ± 8.83	0.90 ± 0.03	0.28 ± 0.01	68.83 ± 0.32	312.42
15	95.32 ± 0.16	96.39 ± 0.12	91.37 ± 0.17	1985.35 ± 13.73	0.82 ± 0.01	0.18 ± 0.01	67.68 ± 0.22	276.53
16	95.32 ± 0.19	77.02 ± 0.07	83.20 ± 0.02	824.54 ± 18.01	0.80 ± 0.01	0.10 ± 0.01	70.18 ± 0.21	148.54
17	95.42 ± 0.15	88.77 ± 0.02	93.66 ± 0.06	2239.50 ± 12.35	0.87 ± 0.01	0.28 ± 0.01	68.67 ± 0.50	302.37

The significance results of the regression equation for each dependent variable are shown in Table A3, with *p* = 0.0026, indicating that the model is highly significant. The lack-of-fit term was not significant, suggesting that the model had a good fit and did not require modification. Therefore, this model could be used to analyze and predict the effects of moisture content, modified PPI addition and MCC addition on the processing characteristics of composite surimi gels. According to the results of the significance tests, the linear term X_1_ and quadratic terms X_1_^2^ and X_3_^2^ had a highly significant effect on the overall score, while the interaction term X_2_X_3_ and the quadratic term X_2_^2^ had a significant effect on the overall score.

**Table A3:** Analysis of variance of the response surface model based on RSM.

Sources of Variation	Sum of Squares	Degree of Freedom	Mean Square	F-Value	*p*-Value	Significance
Model	50,456.23	9	5606.25	10.61	0.0026	**
X_1_	20,320.6	1	20,320.6	38.44	0.0004	**
X_2_	101.95	1	101.95	0.1929	0.6738	
X_3_	73.53	1	73.53	0.1391	0.7202	
X_1_X_2_	0.0244	1	0.0244	0.00	0.9948	
X_1_X_3_	81.11	1	81.11	0.1534	0.7069	
X_2_X_3_	3029.5	1	3029.5	5.73	0.0479	*
X_1_^2^	13,891.01	1	13,891.01	26.28	0.0014	**
X_2_^2^	3659.96	1	3659.96	6.92	0.0339	*
X_3_^2^	6724.22	1	6724.22	12.72	0.0091	**
Residual	3700.47	7	528.64			
Misspecification Loss of fit	2659.97	3	886.66	3.41	0.1335	
Pure error	1040.51	4	260.13			
Sum	54,156.7	16				

* Significant (*p* < 0.05); ** Highly significant (*p* < 0.01).
